# Combined Utilisation of Rapid Assessment Procedures for Loiasis (RAPLOA) and Onchocerciasis (REA) in Rain forest Villages of Cameroon

**DOI:** 10.1186/1475-2883-4-2

**Published:** 2005-04-07

**Authors:** Samuel Wanji, Nicholas Tendongfor, Mathias Esum, Siker SJ Yundze, Mark J Taylor, Peter Enyong

**Affiliations:** 1 University of Buea, Faculty of Science, Department of Life Sciences, P.O. Box 63, Buea, Cameroon; 2 Research Foundation in Tropical Diseases and Environment (REFOTDE), P.O. Box 474, Buea, Cameroon; 3 Filariasis Research Laboratory, Liverpool School of Tropical Medicine, Pembroke Place, Liverpool L3 5QA, UK; 4 Tropical Medicine Research Station, P.O. Box 55, Kumba, Cameroon

## Abstract

**Background:**

Individuals with high microfilarial loads of *Loa loa *are at increased risk of neurologic serious adverse (SAE) events following ivermectin treatment against onchocerciasis. RAPLOA (Rapid Assessment Procedure for loiasis), a newly developed rapid assessment procedure for loiasis that relates the prevalence of key clinical manifestation of loiasis (history of eye worm) to the level of endemicity of the infection (prevalence of high intensity), is a very useful tool to identify areas at potential risk of *L. loa *post ivermectin treatment encephalopathy. In a perspective of treatment decision making in areas of co-endemicity of loiasis/onchocerciasis, it would be advantageous (both in time and cost savings) for national onchocerciasis control programmes to use RAPLOA and the Rapid epidemiologic assessment for onchocerciasis (REA), in combination in given surveys. Since each of the two rapid assessment tools have their own specificities, the workability of combining the two methods needed to be tested.

**Methods:**

We worked in 10 communities of a forest area presumed co-endemic for loiasis and onchocerciasis in the North-West Province of Cameroon where the mass-treatment with ivermectin had not been carried out. A four-step approach was used and comprised: (i) generating data on the prevalence and intensity of loiasis and onchocerciasis in an area where such information is scarce; (ii) testing the relationship between the *L. loa *microfilaraemia prevalence and the RAPLOA prevalence, (iii) testing the relationship between the *O. volvulus *microfiladermia prevalence and the REA prevalence, (iv) testing the workability of combining RAPLOA/REA by study teams in which a single individual can perform the interview for RAPLOA and the nodule palpation for REA.

**Results:**

The microfilaraemia prevalence of loiasis in communities ranged from 3.6% to 14.3%. 6 (0.61%) individuals had *L. loa *microfilarial loads above 8000 mf/ml but none of them attained 30,000 mf/ml, the threshold value above which the risk of developing neurologic SAE after ivermectin treatment is very high. None of the communities surveyed had RAPLOA prevalence above 40%. All the communities had microfiladermia prevalence above 60%. The microfiladermia results could be confirmed by the rapid epidemiologic method (nodule palpation), with all the 10 communities having REA prevalence above 20%. For the first time, this study has demonstrated that the two rapid assessment procedures for loiasis and onchocerciasis can be carried out simultaneously by a survey team, in which a single individual can administer the questionnaire for RAPLOA and perform the nodule palpation for REA.

**Conclusion:**

This study has: (i) Revealed that the Momo valley of the North West province of Cameroon is hyperendemic for onchocerciasis, but is of lower level of endemicity for *L. loa*. (ii) Confirmed the previous relationships established between RAPLOA and the *L. loa *microfilaraemia prevalence in one hand and between the REA and the *O. volvulus *microfiladermia prevalence in another hand (iii) Shown that RAPLOA and REA could be used simultaneously for the evaluation of loiasis and onchocerciasis endemicity in areas targeted by the African Programme for onchocerciasis Control for community-directed treatment with ivermectin (CDTI).

## Background

For several years mass treatment with ivermectin has been used to control onchocerciasis. The community directed distribution of annual doses of ivermectin introduced through the African Programme for Onchocerciasis Control (APOC) is the key component of this programme. In this Community-directed treatment with ivermectin (CDTI), the community itself is in charge of designing and implementing the ivermectin distribution [1].

The mass distribution of ivermectin is always preceded by the mapping of the target area, using rapid epidemiologic mapping of onchocerciasis (REMO), which takes into consideration specific spatio-epidemiological characteristics of onchocerciasis (spatial distribution of vectors in breeding sites along rivers) and the Rapid epidemiologic assessment for onchocerciasis (REA), which is based on the estimation of the prevalence of onchocercal nodules in adult males using simple palpation [2]. The prevalence of palpable nodules in adult males is almost half the prevalence of microfiladermia in the total population (nodule prevalence of 20% corresponds to microfilaria prevalence of 40–60%) [3,4]. Communities with nodule prevalence of 20% and above are eligible for CDTI.

The large-scale distribution of ivermectin was successfully introduced into Cameroon until neurologic serious adverse events (SAEs) in individuals with high *L. loa *microfilaraemia after ivermectin treatment were reported [[5,6] and [7]]. The SAEs are characterized by progressive neurologic decline and encephalopathy within a few days of taking ivermectin. In some cases, this can result in death or chronic disability. A recent retrospective analysis of *L. loa *encephalopathy temporally related to treatment with Mectizan^® ^(PLERM – 'Probable' or 'Possible' *L. loa *Encephalopathy temporally Related to treatment with Mectizan^®^), revealed that 97% of cases so far declared in Africa came from southern Cameroon, with 93% of them being individuals treated with ivermectin for the first time [8]. The main clinical signs and symptoms associated with the PLERM are altered mental status, incontinence, difficulty standing up or walking, dysarthria, fever, diarrhoea, headache and feverishness.

These neurologic SAEs have been observed mainly in areas where *L. Loa *and *O. volvulus *are co-endemic and this has hampered the advancement of the mass treatment with ivermectin in the forested areas. There was therefore an urgent need for a rapid method to identify communities where individuals are at risk of developing neurologic SAEs, before the implementation of mass treatment with ivermectin.

A study carried out in Cameroon and Nigeria in 2001 supported by the UNDP/World bank/WHO Special Programme for Research and Training in Tropical Diseases (TDR) and APOC led to the development of a Rapid Assessment Procedure for loiasis (RAPLOA) [9,10]. From the results of this study it was found that communities in which more than 40% of individuals were reported to have experienced the sub-conjunctival migration of the adult *L. loa*, confirmed by a photograph of the worm in the eye, with the most recent episode lasting between 1–7 days, had a *L. loa *microfilaraemia prevalence of 20% or above. In such communities 5% of individuals harbour more than 8000 mf/ml and are exposed to significant increase risk of occurrence of functional impairment after ivermectin treatment [6]; meanwhile, 2% of them carry more than 30,000 mf/ml and have a risk of serious neurological reactions following ivermectin treatment [5].

It is now recommended by the Mectizan^® ^Expert Committee and the Technical Consultative Committee of APOC (MEC/TCC) that before commencing ivermectin distribution in areas suspected, or known to be endemic for loiasis, RAPLOA should be undertaken to assess the prevalence of *L. loa *[11].

RAPLOA is a newly developed tool and its full programmatic implementation requires that it should be validated, in several independent studies, in sites different from where it was originally developed. Furthermore, it will be advantageous, (both in time and cost savings) for the National onchocerciasis control programmes in Africa, to be able to carry out RAPLOA and REA using single research teams to assess the level of endemicity of the two infections during a given survey. Data from such surveys could be used to facilitate rapid decision making to enable the most effective treatment [12]. For instance, if both RAPLOA and REA are negative, or if RAPLOA is positive and REA is negative, there should be no mass treatment with ivermectin. If REA is positive and RAPLOA is negative, the CDTI can be implemented safely; but if REA is positive and RAPLOA is also positive, mass treatment can be carried out with precautions (possibility of identifying early warning signs of encephalopathy and possibility of therapeutic intervention).

Given that each of the two rapid assessment tools have their own specificities (time factor, mode of assessment, selection of participants and sample size requirements), the workability of combining the two methods, needed to be evaluated. It is in this light that we recently carried out a study in 10 communities, selected from a forest area of North-west Cameroon, an area which has not yet undergone large scale distribution of ivermectin, but which is earmarked for CDTI and where *O. volvulus *and *L. loa *are co-endemic. The general objective of this study was to explore the feasibility of combining RAPLOA and REA in a given survey by a single research team. The specific objectives were to (i) provide information on the prevalence and intensity of loiasis and onchocerciasis in an area where data was scarce, (ii) validate RAPLOA in different sites from where it was originally developed and (iii) to reinforce the validity of nodule palpation in an area of co-endemicity *L. loa*/*O. volvulus*, (iv) to test the workability of combining RAPLOA/REA by study teams in which a single individual can perform the interview for RAPLOA and the nodule palpation for REA.

## Methods

### Study site

**Figure 1:**
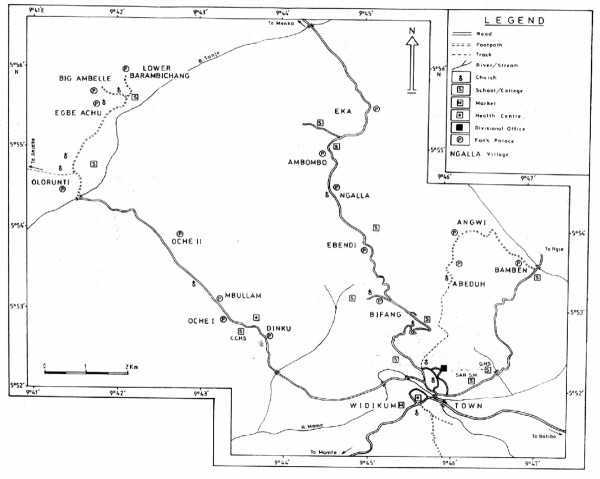
Map of the Widikum area showing the geographical location of the villages surveyed.

The study was carried out in 10 villages located in the Batibo Health District, North West province of Cameroon between March and May 2003. The villages were selected from the Widikum subdivision located in the Momo valley and included: Bifang, Ebendi, Ngalla, Ambombo, Eka, Dinku, Oche1, Oche 2, Mbullam and Olorunti. They extend between latitude 5° N 43 – 5° N 54 and between longitude 9° E 41 – 9° E 44. The vegetation is a degraded forest in which the primary rain forest has been completely replaced by the oil palm, which constitutes the main commercial crop. The inhabitants of these villages are mainly farmers. The climate is tropical with two seasons: the rainy season which lasts from mid March to mid November and the dry season from mid November to mid March. The main rivers are river Momo and Tanjo, which are tributaries of river Manyu (Figure 1).

### Study population

A census was conducted in each of the villages surveyed to estimate the population size. The study population consisted of males and females aged 15 years and above who have been resident in the village for a minimum of five consecutive years and who have not taken antifilarial treatment for a minimum period of one year. All eligible members of the community and who consented to participate were enrolled into the study. The investigation was carried out according to a protocol approved by the ethical committee of the Research Foundation in Tropical Diseases, and Environment, Buea and the Tropical Medicine Research Station Kumba.

### Conduct of the rapid assessment procedures for loiasis and onchocerciasis

#### Organization of work

A form designed to collect data was divided into four sections: the first section was for the identification of participants, the second and third sections for the collection of RAPLOA and REA data with special reference on the starting and ending time for both exercises (RAPLOA and REA). The last section was for the parasitological results of skin snips and thick blood films. In each community surveyed, a team of three technicians moved from one household to another to register eligible participants, administer the RAPLOA questionnaire and carried out a Rapid epidemiological assessment (REA) by nodule palpation. Each participant was then referred to the parasitological post situated at the centre of the village for blood collection and skin snipping.

### Administration of RAPLOA questionnaire

The Rapid Assessment Procedure for loiasis was based on the restricted definition of the eye worm; the past experience of eye worm, confirmed by a photograph of *L. loa *adult worm in the white part of the eye and with the duration of the most recent episode being between 1 to 7 days [9,10]. The questionnaires were administered in the English language and where required, interpreters from the community assisted in the interview process according to the RAPLOA guidelines [13]

### Nodule palpation (REA)

The REA was based on nodule palpation. After undergoing the RAPLOA interview, every patient was examined by the same technician for the presence of *Onchocerca *nodules according to previous studies [4,14].

### Parasitological examinations

These involved the thick blood film to search for *L. loa *microfilariae (mf) and the skin snip for *O. volvulus *microfilariae.

### Thick blood film

The thick blood films were prepared from a standardised 50 μl finger prick blood collected between 10:00 and 16:00 hours using a 75 μl non-heparinised capillary tube [15,16,10].

### Collection and processing of skin snips

Two skin snips were taken with a sterile corneo-scleral punch (Holth 2 mm) from the two iliac crests of each patient. The skin snips were placed in two separate wells of a microtitration plate containing 100 μl of normal saline and covered with parafilm. Between each patient the corneo-scleral punches were sterilised by dipping in hypochlorous solution, followed by 70% ethanol and finally rinsed in distilled water. After 24 hours, a drop of 2% formalin was added to each well to preserve the microfilariae. Microfilariae were counted later using an inverted microscope.

### Expression of results

The results were entered in Epi Info (Version 6.03, 1996) and analysed using SPSS 10.1 for Windows. The RAPLOA prevalence was expressed as the proportion of individuals whose answers to the questionnaire complied with the restricted definition of eye worm. The REA prevalence was expressed as the proportion of individuals with at least one palpable *O. volvulus *nodule.

The arithmetic mean and William's mean were used to express the intensity of the infection in study villages. The arithmetic mean was determined as the average count of mf/ml or mf/skin snip in each village. The William's mean was determined as the geometric mean calculated after having added 1 to each count [17]. The Chi-square test of trend was used to compare prevalence between communities whereas the non-parametric Kruskal and Wallis test was used to assess differences in the intensities of the infection in different communities. Scatter diagrams were plotted with the following cut-off points defined in previous studies. RAPLOA40: Prevalence of loiasis, as determined by the restricted definition of history of eye worm, above which the risk of SAEs post ivermectin treatment is increased. MFLOA20: Prevalence of *L. loa*, determined by thick film method, above which individuals within the community are at risk of SAEs post mass ivermectin treatment. REA20: Prevalence of onchocerciasis, determined by nodule palpation, above which the large-scale treatment with ivermectin is highly desirable. MFDONCHO60: Prevalence of onchocerciasis, determined by skin snipping, above which the large-scale treatment with ivermectin is most urgent. The RAPLOA results generated from this study were superimposed on the same scatter plot with the original data generated during the development of the RAPLOA study for comparison.

## Results

### Prevalence and intensity of loiasis

Table 1 gives the prevalence and intensity of *L. loa *microfilariae as well as the RAPLOA prevalence in study villages. A total of 977 individuals both males and females were enrolled in the study. The prevalence of *L. loa *microfilariae varied from one village to another. The lowest prevalence (3.36%) was observed at Ebendi whereas the highest prevalence (14.29 %) was observed at Mbullam. RAPLOA prevalence also varied from one village to another, ranging from 9.38 % in Oche 1 to 31.03% in Oche 2.

**Table 1:** Prevalence and intensity (mf/ml) of *L. loa *determined by RAPLOA and by the Thick blood film techniques.

			Prevalence (%)		Intensity (mf/ml)			
			
Village	Population*	No. Examined	RAPLOA	Thick blood film	Arithmetic Mean Mf +ve & -ve individuals	Arithmetic Mean Mf +ve individuals	William's means > 15	William's means > 20 (CMFL)
**Ambombo**	250	49	24.49	6.12	1.63	27.67	1.22	1.25
**Bifang**	1889	141	21.99	3.55	81.99	2313	1.25	1.30
**Mbullam**	65	21	19.05	14.29	86.67	608.67	2.36	2.21
**Dinku**	516	159	30.82	10.69	1.3.77	970.79	1.83	2.02
**Ebendi**	260	120	13.33	3.36	8.00	240.00	1.18	1.14
**Eka**	1079	96	14.58	9.38	418.96	4468.89	1.87	1.96
**Ngalla**	765	138	16.67	7.25	232.46	3208.00	1.53	1.55
**Oche 1**	83	32	9.38	12.50	293.13	2345.00	2.08	2.13
**Oche 2**	77	29	31.03	10.34	13.10	126.67	1.609	1.93
**Olorunti**	707	192	20.31	4.69	119.27	2544.44	1.39	1.40
**Total**	**5691**	**977**						
**Average**			**20.47**	**6.86**	**139.08**	**2028.06**	**1.50**	**1.53**

* Total population recorded during a census conducted in study villages; CMFL: Community microfilarial load. **Mf +ve: Microfilaraemic, MF -ve: amicrofilaraemic**

The arithmetic means of *L. loa *in the entire study population ranging from 8 mf/ml of blood in Ebendi to 418.96 mf/ml of blood in Eka. In microfilaraemic individuals they ranged from 27.67 mf/ml in Ambombo to 4468.89 mf/ml in Eka. The William's mean of microfilarial loads also differed from one village to another, ranging from 1.18 mf/ml to 2.36 mf/ml in the entire study population and from 1.14 mf/ml to 2.21 mf/ml in individuals with ≥ 20 years.

### Prevalence and intensity of onchocerciasis

The prevalence and intensity of *O. volvulus *as determined by REA and Skin snips are indicated in Table 2. The prevalence of palpable nodules varied significantly (p < 0.001) from one village to another. These ranged from 20.83% in Eka to 65% in Ebendi. The prevalence of onchocerciasis determined by the skin snips was high in all the 10 villages. It varied from 66.67% at Bifang to 95.24% at Dinku. The William's means varied from 4.08 at Bifang to 17.38 at Dinku. In individuals with age ≥ 20 years, it ranges from 4.19 mf/ml in Bifang to 18.58 in Mbullam.

**Table 2:** Prevalence and Intensity of *O. volvulus *determined by REA and Skin snip

			Prevalence (%)		Intensity (mf/Skin snip)			
			
Village	Population*	No. Examined	REA	Skin snip	Arithmetic Mean Mf +ve & -ve individuals	Arithmetic Mean Mf +ve individuals	William's means ≥ 15	William's means ≥ 20 (CMFL)
**Ambombo**	250	49	40.82	69.39	14.57	21.00	5.07	4.76
**Bifang**	1889	141	38.30	66.67	12.54	18.80	4.06	4.19
**Mbullam**	65	21	23.81	95.24	65.26	68.52	13.38	14.60
**Dinku**	516	159	54.72	94.34	32.45	34.40	17.31	18.58
**Ebendi**	260	120	65.00	85.83	19.70	22.95	7.76	7.82
**Eka**	1079	96	20.83	72.92	14.13	19.37	5.24	5.69
**Ngalla**	765	138	56.52	76.09	18.45	24.25	6.85	7.70
**Oche 1**	83	32	21.88	87.50	11.25	12.86	5.41	6.52
**Oche 2**	77	29	55.17	86.21	28.07	32.56	10.53	10.49
**Olorunti**	707	192	39.06	86.46	31.68	36.43	10.57	10.89
**Total**	**5691**	**977**						
**Average**			**45.04**	**81.37**	**23.06**	**28.31**	**7.99**	**8.22**

* Total population recorded during a census conducted in study villages, CMFL : Community microfilarial load ; **Mf +ve: Microfiladermic, MF -ve: amicrofiladermic individuals & -ve**

### Knowledge of eye worm, attitude and practice in study communities

The eye worm was well known in these communities. In Bifang, Ebendi, Ngalla, Ambombo and Eka it is called "Embele", in Dinku, Oche 1, Oche 2 and Mbullam it is called "ntembele". In Olorunti it is called "etembele". These names were generally descriptive. It was noticed that communities have "Eye Worm specialists", who are capable of removing adult *L. loa *as they migrate across the subconjunctiva.

### Relationship between the RAPLOA and *L. loa *microfilaraemia prevalence

**Figure 2:**
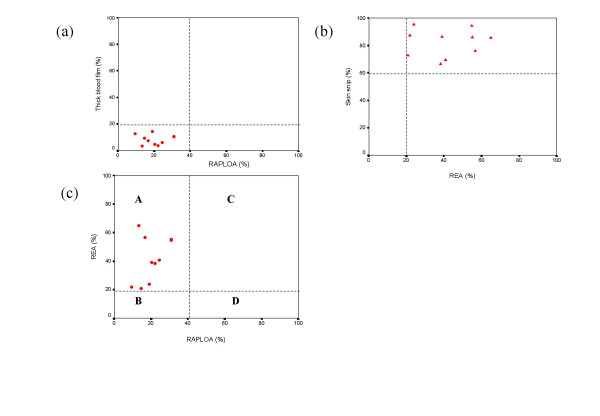
**a**: Relationship between the RAPLOA and *L. loa *microfilaraemia prevalences. Dotted lines represent the thresholds levels above which there is increased risk of neurologic SAEs. **b **Relationship between the REA and the microfiladermia prevalences. Dotted lines represent the thresholds levels above which mass treatment with ivermectin is most urgent (microfiladermia) or highly desirable (REA). **c **Relationship between RAPLOA and REA prevalences. **A: **Large-scale treatment with low risk of neurologic SAEs; **B: **Large scale treatment with high risk of neurologic SAEs; **C: **No large-scale treatment with ivermectin **D: **No large-scale treatment with ivermectin. Dotted lines represent the thresholds levels above which the risk of neurologic SAEs is high

**Figure 3:**
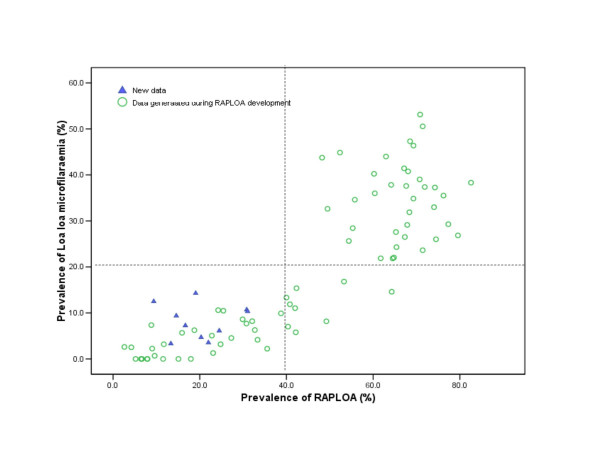
Relationship between RAPLOA prevalence and Loa microfilaraemia: (▲) New data, (o) Data generated during RAPLOA development.

Figure 2a summarises the relationship between the parasitological prevalence and RAPLOA prevalence. All the villages surveyed had RAPLOA prevalences less than 40%. *L. loa *microfilaraemia prevalences were less than 20 % in all the communities. The parasitological prevalences are in agreement with the RAPLOA predictions. These results fitted very well when superimposed on the original data generated during the development of RAPLOA (Figure 3).

### Relationship between the REA prevalence and microfiladermia prevalence

Figure 2b summarises the relationship. All the communities surveyed had REA prevalence above 20% and prevalence of microfiladermia greater than 60%. On average, the skin snip prevalences (81.37%) were nearly twice as high as the REA prevalences (45.04%). There is good agreement between the two sets of prevalence, indicating that the study area has high endemicity for onchocerciasis. Some communities with REA close to 20% had high microfiladermia prevalence (> 70 %).

### Combining RAPLOA and REA

This study has revealed that an examiner, using a single recording form with specific sections for REA and RAPLOA can take 8–10 minutes to examine an individual for loiasis and onchocerciasis in tandem. With three technicians performing the two tasks at the household's level, it took on average 4 hours to cover one community with a sample size of 80 people per day. Not-withstanding the difficulties of movement in the study area due to bad roads or lack of passable roads, we could examine during this survey on average two communities with an average sample size of 80 individuals per day. In the village Olurunti, working from 10:00 AM to 3:00 PM we examined 192 individuals. Figure 2c summarises the relationship between REA (%) and RAPLOA (%). All the communities surveyed had REA above 20% and RAPLOA less than 40% indicating that CDTI could be conducted within them safely.

## Discussion

### Endemicities of onchocerciasis and loiasis in the study area

This study has revealed that the Widikum health area situated in the Momo valley is an area hyperendemic for onchocerciasis. All the communities surveyed had microfiladermia prevalences above 60%. The intensity of infection as expressed by the arithmetic means and the William geometric means were relatively low. None of the communities had an arithmetic mean above 100 mf/skin snip. This lack of close relationship between the prevalence and intensity of infection could be explained by the existence of passive treatment with ivermectin in the area. Even though the CDTI was not in place in this area at the time of this survey, it could be noticed that almost 25% of individuals involved in the study stated they had taken ivermectin 2 to 3 years before the study (*unpublished observations*). Since the effect of ivermectin can last up to 3 years, this could have contributed to the reduction of microfilarial density. Indeed, the Momo valley is close to the South-West Province where the CDTI has been going on for over 5 years and some people admitted getting treatment from the South-West project.

The infection level of *L. loa *was relatively low. None of the communities surveyed had *L. loa *microfilariae prevalence (mf-prevalence) greater than 20%. According [16], for practical purposes, communities with *L. loa *mf-prevalence less than 10% should be considered as hypo endemic and communities with mf-prevalence between 10% and 20% should be considered as meso-endemic. The intensity of *L. loa *infection in microfilaraemic patients as expressed by the arithmetic mean did not exceed 4500 mf/ml, contrasting with the observations made in the Lekié division [16], Central Province of Cameroon, where some communities examined for *L. loa *had intensities up to 22791 mf/ml of blood. It should be noted that the Lékié division is the area where most of the cases of *Loa*-related encephalopathies were observed. The results of this study also contrast with the observations made in the Ntem valley (a forested savannah zone in the North-west province of Cameroon) [17], where in a survey carried out in10 communities, *L. loa *intensities (arithmetic mean of total number of patients examined) of up to 9995 mf/ml were observed in some communities. However, the findings of the present study were very similar to the observations made in the forest villages of south-west Province of Cameroon [18].

### Relationship between the Rapid assessment procedure prevalences and the parasitological prevalences

#### – RAPLOA prevalence *versus *Loa-microfilaria prevalence

None of the communities surveyed had RAPLOA prevalence above 40% (threshold value established by the RAPLOA development study) [10]. Also none of the communities had *L. loa *microfilareamia above 20% (threshold prevalence above which more than 5% of adults living in the community are at risk of adverse-related responses with ivermectin) [16]. There is therefore a good agreement between the results from the Thick blood film technique and the results from RAPLOA, since the two sets of findings have reached the same conclusion that the study area is not hyperendemic for loiasis and that the risk of serious adverse events after ivermectin treatment may be very low in these communities. In this light, this independent study has validated the rapid assessment procedure for loiasis (RAPLOA) in predicting the level of endemicity of loiasis and the risk of neurologic serious adverse events after ivermectin treatment in a given area. It may not be necessary therefore to strengthen monitoring by a medical team, after a campaign of mass treatment with ivermectin. Indeed, a mass treatment with ivermectin, carried out in the 10 communities and involving some 4000 eligible individuals did not show any neurologic serious adverse events related to ivermectin treatment (*unpublished data*)

### - REA prevalence versus *Onchocerca *microfiladermia prevalence

All the villages surveyed had prevalences of palpable nodules above 20% (threshold defined as proportion above which a community should be included in the CDTI programme). These REA results were confirmed by skin snip results; all the communities examined had microfiladermia prevalences above 60%. These two sets of results confirm that the Momo valley, characterized by important fast flowing rivers with excellent breeding sites for *Simulium damnosum s.l*, is an area of high endemicity of onchocerciasis. The results of this study are in agreement with previous studies [4,14] and constitute a further validation of the REA to predict the level of endemicity of onchocerciasis. Although the validation of REA has been done before, this is the first time such validation is being done in an area of co-endemicity of onchocerciasis/loiasis.

### Combining REA and RAPLOA and its implications for the onchocerciasis control programmes' activities in Africa

The recent MEC/TCC recommendation that before introducing the mass distribution of ivermectin for onchocerciasis control in an area suspected or known to be endemic for loiasis, RAPLOA should be carried out to assess the prevalence of *L. loa*, has necessitated the evaluation of the possibility of combining REA and RAPLOA, the two assessment procedures for onchocerciasis and loiasis endemicity.

The two tools have several similarities in their methodological approaches: they are carried out on individuals (adults or nearly-adults) of both sexes, who have been resident in a community for a long period of time, their sample size requirements are not conflicting, the exercises of interview and nodule palpation are very simply to execute. However, the question of whether a single examiner could perform the two tasks in succession, with a high level of efficiency, as measured by the ratio output/time, arose. In other words, how long can it take an examiner to assess a patient for the two filarial infections? This study has shown that it can take 8 to 10 minutes. By extrapolating this basic result to a survey team made up of 5 individuals who can carry out the two procedures in tandem, it is possible to examine 80 subjects in less than three hours. This will allow a good team to evaluate 3–4 communities per day under optimum conditions (good mobilisation, community accessibility). Indeed, we were able in the present study to examine 192 individuals in the village of Olurunti from 10:00 AM to 3: 00 P.M with a team of 3 persons.

The possibility of simultaneously carrying out at the community level RAPLOA and REA for assessing loiasis and onchocerciasis prevalences will have a great impact on the planning and implementation of activities of onchocerciasis control programmes in areas of co-endemicity with loiasis. There will be a gain both in time and in cost savings, since a single well organized survey in a given area will be enough to assess the level of endemicity of the two filarial species. More important will be the fact that, data from such surveys could be used rapidly in treatment decision making. For instance, if both RAPLOA and REA are negative (prevalence <40% and <20% respectively), or if RAPLOA is positive (prevalence >40%) and REA is negative, there should be no mass treatment with ivermectin. If REA is positive (prevalence >20%) and RAPLOA is negative, the CDTI can be implemented safely. But if REA is positive and RAPLOA is also positive, mass treatment can be carried out following MEC/TCC guidelines. The results of this combined survey RAPLOA/REA clearly indicate that the CDTI could be implemented safely in the Momo valley of the North-west province of Cameroon. Indeed, a CDTI project, which is now in its second year of activities in this area, has not so far registered any neurologic SAE (*unpublished data*).

## List of Abbreviations

APOC: African Programme for Onchocerciasis Control

CDTI: Community-Directed Treatment with Ivermectin

SAEs: Severe Adverse Effects

MEC/TCC: Mectizan Expert Committee/Technical Consultative Committee

REMO: Rapid Epidemiological Mapping of Onchocerciasis

CMFL: Community Microfilarial Load

RAPLOA: Rapid Assessment Procedure for Loiasis

RAPLOA40: Loiasis prevalence, as determined by RAPLOA, above which the risk of serious adverse effect is increased

MFLOA20: Loiasis prevalence, as determined by thick blood film method, above which the risk of serious adverse effect is increased

REA: Rapid Epidemiological Assessment of Onchocerciasis

REA20: Onchocerciasis prevalence, as determined by REA, above which the large-scale treatment with ivermectin is highly desirable

MFD-ONCHO60: Onchocerciasis prevalence, as determined by skin snipping, above which the large-scale treatment with ivermectin is most urgent

Mf-prevalence: microfilarial prevalence

PLERM: 'Probable' or 'Possible' Loa Loa Encephalopathy temporally related to treatment with Mectizan

## Authors' contributions

**SW: **Participated in the design of the study, collection and processing of the data, drafting and editing of the manuscript.

**TN: **Participated in the collection, processing and analysis of data.

**YSSJ: **Participated in the collection and processing of data.

**EM: **Participated in the collection and processing of the data

**MJT: **Collection of data and review of the manuscript,

**EP: **Participated in the design of the study, data collection and review of the manuscript.
